# Year-round breeding equatorial Larks from three climatically-distinct populations do not use rainfall, temperature or invertebrate biomass to time reproduction

**DOI:** 10.1371/journal.pone.0175275

**Published:** 2017-04-18

**Authors:** Henry K. Ndithia, Kevin D. Matson, Maaike A. Versteegh, Muchane Muchai, B. Irene Tieleman

**Affiliations:** 1 Ornithology Section, Department of Zoology, National Museums of Kenya, Nairobi, Kenya; 2 Groningen Institute for Evolutionary Life Sciences, University of Groningen, Groningen, the Netherlands; Liverpool John Moores University, UNITED KINGDOM

## Abstract

Timing of reproduction in birds is important for reproductive success and is known to depend on environmental cues such as day length and food availability. However, in equatorial regions, where day length is nearly constant, other factors such as rainfall and temperature are thought to determine timing of reproduction. Rainfall can vary at small spatial and temporal scales, providing a highly fluctuating and unpredictable environmental cue. In this study we investigated the extent to which spatio-temporal variation in environmental conditions can explain the timing of breeding of Red-capped Lark, *Calandrella cinerea*, a species that is capable of reproducing during every month of the year in our equatorial east African study locations. For 39 months in three climatically-distinct locations, we monitored nesting activities, sampled ground and flying invertebrates, and quantified rainfall, maximum (T_max_) and minimum (T_min)_ temperatures. Among locations we found that lower rainfall and higher temperatures did not coincide with lower invertebrate biomasses and decreased nesting activities, as predicted. Within locations, we found that rainfall, T_max_, and T_min_ varied unpredictably among months and years. The only consistent annually recurring observations in all locations were that January and February had low rainfall, high T_max_, and low T_min_. Ground and flying invertebrate biomasses varied unpredictably among months and years, but invertebrates were captured in all months in all locations. Red-capped Larks bred in all calendar months overall but not in every month in every year in every location. Using model selection, we found no clear support for any relationship between the environmental variables and breeding in any of the three locations. Contrary to popular understanding, this study suggests that rainfall and invertebrate biomass as proxy for food do not influence breeding in equatorial Larks. Instead, we propose that factors such as nest predation, female protein reserves, and competition are more important in environments where weather and food meet minimum requirements for breeding during most of the year.

## Introduction

Ecological and environmental factors, such as food availability and weather, shape reproductive decisions in many bird species. These factors can act alone or in combination, and they may fluctuate in predictable or unpredictable ways within and between years. For birds living at mid-latitude locations in the temperate zone, predictable seasonal changes in day length and other environmental conditions function as reproductive cues. These birds can potentially time reproduction with regularity [1, 2, 3, 4]. Increasing photoperiod, a characteristic of temperate zone spring, triggers reproduction in birds via neuroendocrine mechanisms [5, 4, 6]. Food availability and temperature serve as supplementary cues to fine-tune the timing of reproduction to local environmental conditions [7, 8, 5, 4]. The net result is synchronized spring breeding within and among species living at the same location [7, 5].

In contrast, birds living in equatorial locations experience little predictable intra-annual variation in day length [9, 10], but instead experience large, and frequently unpredictable, variation in rainfall and food availability [11, 12, 13, 10, 14, 15, 16, 17, 18]. By living in environments without predictable seasonal cues, equatorial birds are thought to time reproduction based on shorter-term and more irregular factors, such as rainfall and food availability [19, 15]. Additionally, these birds tend to have more flexible breeding schedules and may breed opportunistically [12, 10]. For example, initiation of breeding with the onset of rain [19, 20, 21] greatly promotes nesting success in low-latitude birds [22, 23, 24]. Likewise in some equatorial birds, timing of reproduction coincides with peaks in food supply [19, 21, 25, 2, 14, 17]. These observations that in unpredictable equatorial environments birds preferentially breed at times of the year with higher rainfall and food, match with the general pattern that environments that are more arid have lower primary productivity and select for reduced reproductive effort [26, 27]. Other factors, such as wind and mist, both of which effectively lower ambient temperature, may also be important [28, 29].

One possible consequence of commencing breeding in response to unpredictable localized conditions is that a single species living in distinct environments might show variation in breeding patterns on a small geographical scale [17]. Exploiting such small-scale variation in environmental aridity within the tropics, we intensively investigated year-round breeding in three equatorial populations of Red-capped Larks *Calandrella cinerea* (Gmelin 1789) in Kenya for 39 consecutive months. These resident populations, despite their close geographic proximity, experience different patterns of temperature and rainfall, with climates ranging from warm and dry to cool and wet, and representing an expected gradient of increasing primary productivity [30]. Thus, the study system allows for a comparative, intraspecific analysis of environmentally induced spatio-temporal variation in reproduction. Rarely have studies assessed year-round breeding activities of equatorial species and related breeding to biotic and abiotic characteristics of climatically-distinct locations.

The overall objective of our study was to compare and understand breeding in Red-capped Larks in relation to spatio-temporal variation in weather conditions and food resources. Specifically, we 1) compared spatial variation in rainfall, temperature, invertebrate biomass and breeding across our three study locations, 2) described within-location year-round patterns of rainfall, temperature and invertebrate biomass and how these variables co-vary with breeding and, 3) determined which, if any, biotic and abiotic factors are related to occurrence and intensity of breeding by Red-capped Larks in each location. We predicted that the drier and warmer the location, or the drier and warmer the time of the year, the lower the productivity of invertebrates and the lower the intensity of breeding by Larks.

## Materials and methods

### Study system

Red-capped Larks are small grassland birds that are widely distributed across Africa. They prefer habitats dominated by short grasses or almost bare ground, including fallow and cultivated agricultural fields. Red-capped Larks feed mostly on invertebrates (including beetles, wasps, caterpillars, butterflies and moths, earthworms, and grasshoppers) and occasionally on grass seeds (pers. obs.). Pairs build ground-level open-cup nests that are placed next to a scrub or grass tuft. They typically lay two eggs per clutch (mean 1.89 ± 0.33 (SD) eggs, n = 279, range 1–3 eggs; pers. obs.). During breeding, birds defend the area around the nest but neighboring nests can be as close as 10 m; outside breeding they occur in flocks (pers. obs.). Before our study, nothing had been documented about timing, number of breeding attempts and other breeding parameters at the individual or population level.

From January 2011 to March 2014, we worked simultaneously in multiple plots in South Kinangop, North Kinangop and Kedong (see Table 1 for details per plot), three locations in central Kenya with distinct climates. Distances between locations are 19 km (South Kinangop—North Kinangop), 29 km (South Kinangop—Kedong) and 34 km (North Kinangop–Kedong). Accessible plots within locations were chosen based on observations of Red-capped Larks made by local bird watchers and by us (H.K.N., B.I.T.). We set up a weather station (Alecto WS-3500, Den Bosch, Netherlands) at each location (Table 1) to measure daily rainfall (mm) and minimum (T_min_) and maximum (T_max_) temperatures (^°^C). Using these measurements from three full calendar years (March 2011 –February 2014), we calculated annual and monthly rainfall, and annual and monthly T_min_ and T_max_.

**Table 1:** Coordinates, altitude (m ASL), surface area (km^2^) and distance to weather station for each plot in our three study locations South Kinangop, North Kinangop and Kedong.

Location	Plot name (altitude, m ASL) and coordinates	Plot surface area (km^2^)	Distance to weather station (km)
South Kinangop	Kenyatta road (2679); 0^0^49’23”S, 36^0^34’39”E	0.3	18.9
	Sasumwa (2508); 0^0^45’03”S, 36^0^39’22”E	0.2	9.4
	Seminis (2556); 0^0^42’30”S, 36^0^36’30”E	1.2	5.2
North Kinangop	Joshua (2451); 0^0^36’00”S, 36^0^28’27”E	0.2	3.8
	Mbae (2425); 0^0^36’54”S, 36^0^30’48”E	0.35	2.5
	Ndarashaini (2412); 0^0^34’33”S, 36^0^29’41”E	0.3	1.8
Kedong	A (2064); 0^0^53’07”S, 36^0^24’32”E	0.5	7.3
	B (2075); 0^0^52’45”S, 36^0^23’29”E	0.4	10.4
	C (2076); 0^0^53’37”S, 36^0^23’54”E	0.9	9.6
	D (2075); 0^0^53’44”S, 36^0^24’32”E	0.9	6.8

South and North Kinangop lie on a plateau of montane grassland along the Aberdare mountain ranges. Study plots in South and North Kinangop flood periodically during rains and standing water remains after rains have stopped (pers. obs. 2010–2014). In South Kinangop, birds bred only in Seminis, despite initially observing them also in the other two plots (Table 1). Flooding made Seminis unavailable for breeding from April–December 2012 and April 2013. Flooding in North Kinangop affected nests located in parts of Joshua and Ndarashaini in October 2011 and October 2012; these plots also received heavy rainfall in April 2013 that affected nesting activities. Kedong, a privately owned ranch in the Rift Valley in Naivasha, consists of large grassland patches that did not flood (pers. obs. 2010–2014).

The study species involved is not and endangered or protected species. The National Museums of Kenya approved this research and owners of the land gave permission to conduct the study on their respective sites.

### Estimating invertebrate availability

To assess invertebrate biomass as proxy for food availability in each location, we used pitfall traps and sweep nets to sample ground and flying invertebrates respectively once per month [31]. We assessed within-location variation in invertebrate biomass using data from plots within a location. For pitfall traps, we used plastic cups with a 26 cm circumference that contained ≈100 ml of 5% formaldehyde solution and that we buried so that rims of the cups were at ground level. We placed five pitfall traps in each plot and left them in place for five days each month. We placed the traps 70 m apart along a 280m-long transect in all plots except one plot in South Kinangop (Seminis), where instead we equally spaced 10 pitfall traps along a 630m-long transect. For sweep-netting (net diameter 0.4m), we established permanent 50m long transects in each plot, subjectively selected as representative for the plot. Per location, one field assistant collected invertebrates between 9:00–10:00 am. If it rained during this hour, we postponed sweep-netting to the same hour on a day without rain. All field assistants were trained to sample in the same manner. We standardized the analyses of pitfall and sweep net sampling data per location (see statistical analysis section below). To calculate annual average and monthly average biomasses, we used two complete calendar years (24 months, March 2011-February 2012 and March 2013-February 2014), excluding the year in which flooding caused multi-month gaps in the data (March 2012-February 2013). Our resulting 24-month data sets had three missing values for ground invertebrates (October 2013, North Kinangop; October and December 2011, Kedong) and three missing values for flying invertebrates (October 2011, October 2013, North Kinangop; February 2014, South Kinangop). For calculations of annual and monthly averages, we substituted each missing value with the average value of the preceding and subsequent months.

We preserved collected specimens in 70% alcohol, later identifying and sorting them based on morphology [32]. For biomass estimation, we classified invertebrates into 10 categories based on size and shape: ants; bees and wasps; beetles and bugs; butterflies and moths; caterpillars, caddisflies, and stoneflies; diplura, millipede, centipede, and earthworms; flies; grasshoppers, crickets, and mantis; spiders, ticks, and mites; and the rest (woodlice, cicadas, cockroaches and earwigs).

We estimated biomass of each of our invertebrate categories as a proxy for food availability. To do this, we first used a subsample of 2198 invertebrate specimens, representing all invertebrate categories from all locations, to develop a category-specific calibration curve relating dry mass as a function of length and width [31, 33, 34]. For every individual in the subset, we measured length (anterior-most part of the head to the tip of abdomen) and width (the widest point of abdomen) using vernier calipers, dried them in an oven for 48 hours at 65°C [35, 33, 34], and measured dry mass on an analytical balance (model KERN ACS 220-4N, KERN and Sohn of Belingen, Germany). We used a log-transformed power model to describe the length-width-mass relationship; the power model has been shown to give the highest adjusted r^2^ compared to length-mass and length-area relationships [35, 33, 34]:

biomass = *a* + *b* log(length) + *c* log (width), where *a*, *b* and *c* are coefficients of the model from each of the invertebrate categories whose biomass we estimated

We used calibration curves per invertebrate category to predict body mass from length and width (for details on the adjusted r^2^ and the range of length and width, see S1 Appendix).

Overall, we collected, measured, dried, and applied the biomass estimation protocol to 23,628 specimens from pitfall traps and 3260 captured by sweep-netting (including calibration subset).

### Lark reproduction

To determine the year-round breeding activities of Larks, we spent on average 134 person-hours per month searching for nests in the three locations combined (Table 2 provides a breakdown for effort per location). Our nest search strategy included observing breeding behavior (e.g., transport of nest materials or food, breeding-related alarm calls, nervous parental behavior around nest sites) and routinely walking plots to flush parents incubating eggs or brooding young. We quantified nest-searching effort as person-hours, i.e., number of hours searching for nests multiplied by the number of persons searching. For each month we calculated a “nest index” (i.e., level of breeding) value, which we defined as the total number of nests found in a month per 10 person-hours of effort. To calculate annual average and monthly average nest indices we used the two complete calendar years (24 months) of March 2011-February 2012 and March 2013-February 2014, excluding the year in which flooding caused multi-month gaps in the data (March 2012-February 2013). Our resulting 24-month data set had one missing value (April 2013, South Kinangop), for which we substituted the average of March and May 2013.

**Table 2:** Search effort (in days (days had a minimum of 2 searching hours) and hours per month) for nests of Red-capped Larks *Calandrella cinerea* in our three study locations South Kinangop, North Kinangop and Kedong, from January 2011 to March 2014.

	Search effort (days/month)		Search effort (hours/month)	
Location	Average + SD	Range	Average + SD	Range
South Kinangop	6.6 ± 2.94	1–13	43.9 ± 24.24	3–130
North Kinangop	8.6 ± 2.20	3–13	40.3 ± 17.06	7–87
Kedong	14.1 ± 5.30	7–24	49.8 ± 35.95	17–193

### Statistical analyses

For all analyses, we tested and confirmed that the dependent variable and the final models observed the assumptions of normality and homoscedasticity of variance through graphical and statistical methods. We tested for among-location differences in rainfall, T_min_ and T_max_, ground and flying invertebrates, and nest index (continuous variable) using mixed models (R-package lme) with location as fixed factor and month as random factor. To compare invertebrate biomasses among plots and locations, we standardized ground and flying invertebrate sampling by expressing biomass per five pitfall traps and one sweep net session per plot or location per month. For among-location comparisons, we log-transformed ground and flying invertebrate data because they were not normality distributed. We found no significant among-plot differences in ground invertebrate biomasses within South Kinangop (F_2, 47_ = 0.89, P = 0.42) or in Kedong (*X*^2^ = 3.98, P = 0.26). For North Kinangop, among-plot differences in ground invertebrate biomasses were significant (*X*^2^ = 6.49, P = 0.04), although post-hoc tests showed no significant differences among-plots. There were no significant among-plot differences in flying invertebrate biomasses for any of the three locations (all *X*^2^ < 5.74, P > 0.06). Therefore, we used the mean monthly biomass per location to test for among-location differences.

We investigated if and how environmental conditions in the month before breeding (“prior”) and in the month of breeding (“current”) were associated with the occurrence and intensity of breeding. We calculated pairwise correlation coefficients between the environmental factors per location (supplementary material 2), to identify potential collinearity. We used model selection based on the Akaike Information Criterion corrected for sample size (AICc) because this allows for exploration of multiple models simultaneously (Burnham and Anderson 2002). To investigate what determines occurrence of breeding, we transformed the continuous variable nest index into the new variable “occurrence of breeding” (binomial: presence/absence). We then used generalized linear mixed models with a binomial distribution to construct for each location a “full” model with the new dependent variable “occurrence of breeding”. These full models included ten explanatory variables (i.e., prior and current rain, T_min_, T_max,_ ground invertebrate biomass, and flying invertebrate biomass) and four two-way interactions, i.e., prior and current rain and corresponding ground invertebrate biomass and prior and current rain and corresponding flying invertebrate biomass. We compared all the possible models and ranked them in order of their AICc, such that the lowest values were considered to have more statistical power [36]. The model with the highest weight and the lowest AICc value was considered the most parsimonious, although all models within 2 AICc of the best model were included in further analysis (Grueber et al. 2011). We explored the relative contribution of the various environmental parameters to breeding by applying model averaging and standardization based on all models with ΔAICc values < 2 (the “best model-set”), compared with the top model (Grueber et al. 2011). Although AICc values of models in the best model-set without month as random effect were higher than models with random effect, we added month as a random effect to the models to correct for potential seasonal effects. We analyzed all data using R statistical software (version 3.0.3) [37].

In the second part of our analysis we investigated how environmental conditions in the month before breeding and in the month of breeding were associated with the intensity of breeding. For this, we analyzed only the months in which breeding occurred (i.e. nest index > 0). We used linear models with a Gaussian distribution and constructed a “full” model with continuous variable “nest index”, for each location. We used the same explanatory variables and statistical approach as in the analysis above. Because month never improved the models in the analysis of occurrence of breeding (see above), and in order to maximize power for the tests of the effects of environmental factors we did not include month as a random effect in these models. In addition, because of low sample size in South Kinangop (n = 7 months), we performed the analyses of intensity of breeding only for North Kinangop and Kedong.

Additional analyses, in which we explored the effects of different time windows and time lags of the environmental variables (up to six months preceding breeding) on breeding occurrence and intensity, did not result in qualitatively different results (see supplementary material 1).

## Results

### Spatial differences in environmental factors, invertebrates and breeding of Larks

**Fig 1:**
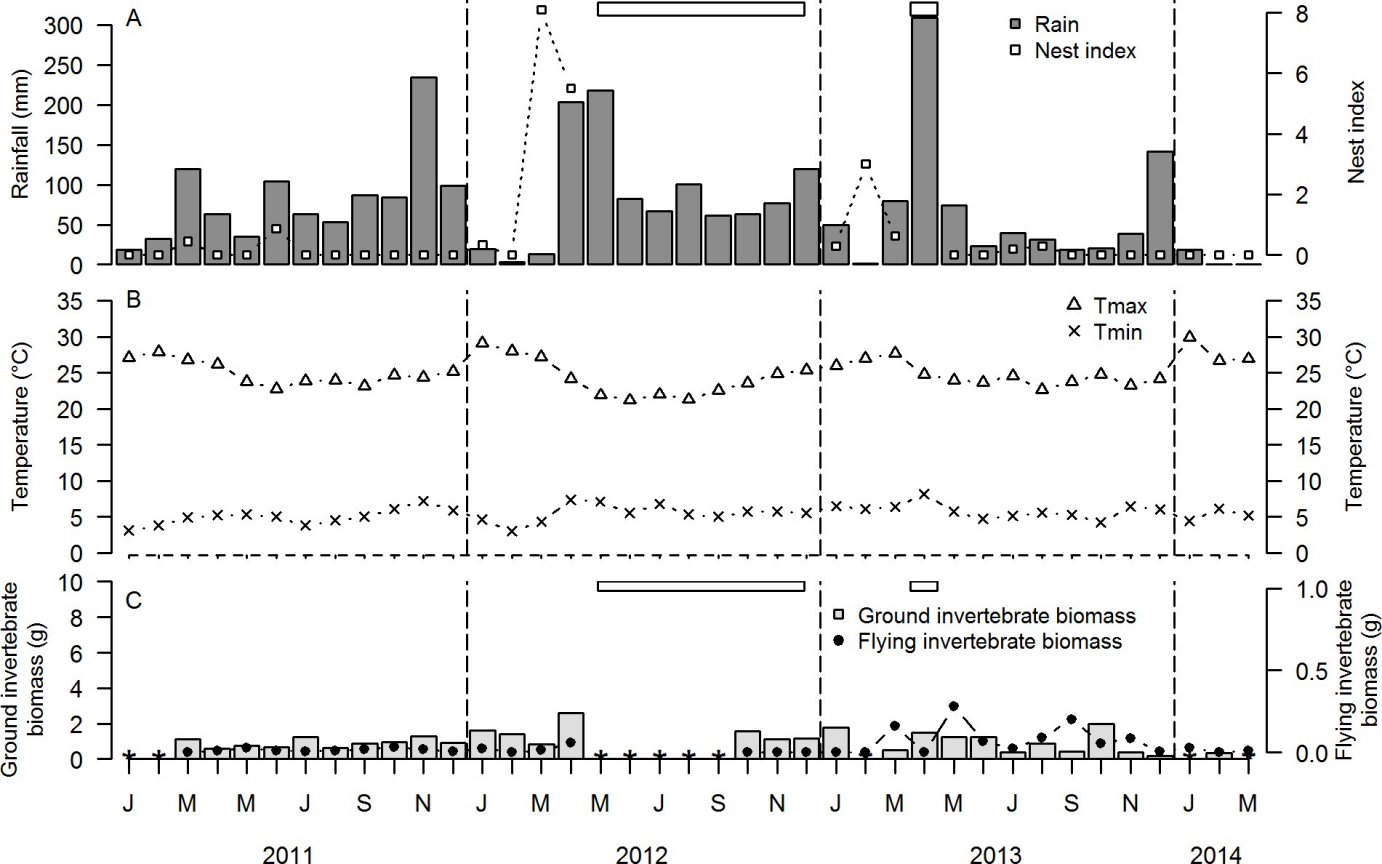
Temporal variation during January 2011-March 2014 of A. rainfall (mm) and nest index (number of nests/10 search h) of Red-capped Larks *Calandrella cinerea*, B. average monthly maximum (T_max_) and minimum (T_min_) temperature (°C), C. biomass (g dry weight) of ground-dwelling and flying invertebrates in South Kinangop. Horizontal open rectangles represent periods of flooding (i.e. standing water in the study location). Data gaps other than from flooding in ground and flying invertebrates represent missing data due to e.g., vandalism (see Methods).

**Fig 2:**
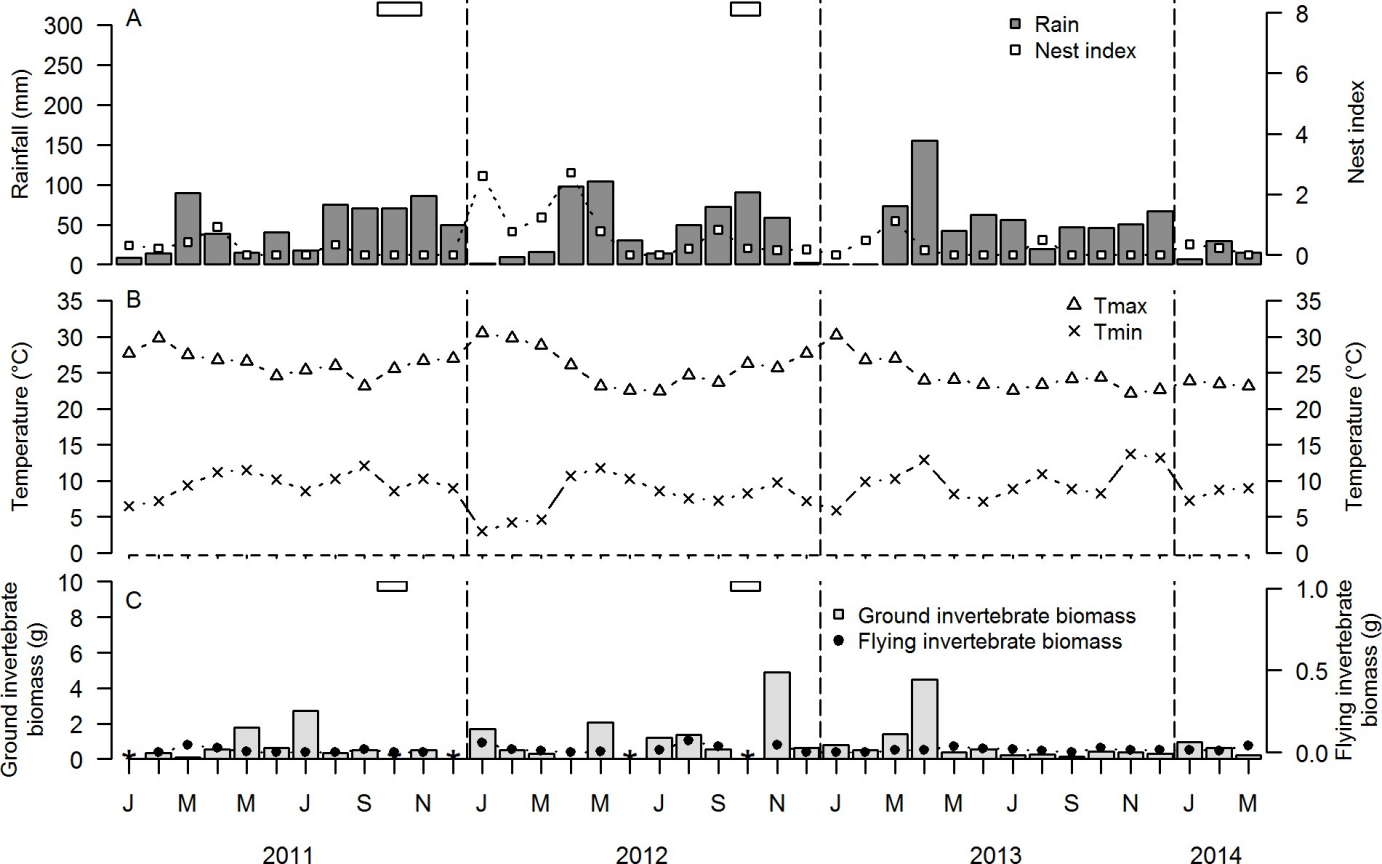
Temporal variation during January 2011-March 2014 of A. rainfall (mm) and nest index (number of nests/10 search h) of Red-capped Larks *Calandrella cinerea*, B. average monthly maximum (T_max_) and minimum (T_min_) temperature (°C), C. biomass (g dry weight) of ground-dwelling and flying invertebrates in North Kinangop. Horizontal open rectangles represent periods of flooding (i.e. standing water in the study location). Data gaps other than from flooding in ground and flying invertebrates represent missing data due to e.g., vandalism (see Methods).

**Fig 3:**
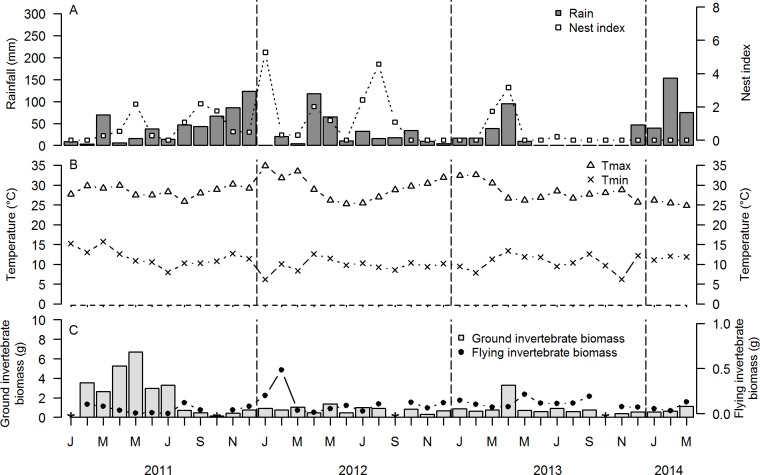
Temporal variation during January 2011-March 2014 of A. rainfall (mm) and nest index (number of nests/10 search h) of Red-capped Larks *Calandrella cinerea*, B. average monthly maximum (T_max_) and minimum (T_min_) temperature (°C), C. biomass (g dry weight) of ground-dwelling and flying invertebrates in Kedong. Data gaps in ground and flying invertebrates represent missing data due to e.g., vandalism (see Methods).

Mean annual and monthly rainfall were highest in South Kinangop, intermediate in North Kinangop and lowest in Kedong (Table 3; Figs 1A, 2A and 3A). South Kinangop received on average 123% more rain than Kedong, while North Kinangop received 40% more rain than Kedong. T_min_ and T_max_ were lowest in South Kinangop (annual average T_min_ = 5.5°C, annual average T_max_ = 24.7°C), intermediate in North Kinangop (annual average T_min_ = 9.1°C, annual average T_max_ = 25.4°C) and highest in Kedong (annual average T_min_ = 10.5°C, annual average T_max_ = 28.6°C) (Table 3; Figs 1B, 2B and 3B).

**Table 3:** Annual (n = 3 years) and monthly (n = 36 months) rainfall (average ± SD, and range), and monthly minimum and maximum temperatures (n = 36 months, average ± SD, and range) as measured by our weather stations in South Kinangop, North Kinangop and Kedong, during March 2011 –February 2014.

Location	Annual rainfall (mm)	Monthly rainfall (mm)		Monthly minimum temperature (^0^C)		Monthly maximum temperature (^0^C)	
	Mean ± SD	Mean ± SD	Range	Mean ± SD	Range	Mean ± SD	Range
S. Kinangop N. Kinangop Kedong	939 ± 132.7 584 ± 62.6 419 ± 96.8	78 ± 69.7^a^ 49 ± 35.3^b^ 35 ± 39.2^b^	0–309 0–155 0–153	5.5 ± 1.06^a^ 9.1 ± 2.42^b^ 10.5 ± 1.92^c^	3.0–8.2 3.0–13.7 6.2–15.7	24.7 ± 2.09^a^ 25.4 ± 2.27^a^ 28.6 ± 2.44^b^	21.2–30.0 22.1–30.5 25.3–34.9

Superscripts indicate subsets of significant differences (P<0.05) among locations in post-hoc tests, after mixed-model analyses. Note: This table contains data for three complete calendar years, but data used for analyses of breeding (Figs 1, 2 and 3) comprise the entire study period of January 2011 –March 2014.

Biomasses of ground invertebrates (log pitfall, mg) did not differ significantly among locations, but biomasses of flying invertebrates (log sweepnet, mg) were highest in Kedong, intermediate in South Kinangop, and lowest in North Kinangop (Table 4). Flying invertebrate biomasses were on average 42% lower in North Kinangop than in Kedong and 27% lower in South Kinangop than in Kedong; flying invertebrate biomasses did not differ significantly between South and North Kinangop (Table 4).

**Table 4:** Annual and monthly biomass of ground invertebrates (log(mg dry mass/5 pitfalls)) and flying invertebrates (log(mg dry mass/sweep-net session)) in South Kinangop, North Kinangop and Kedong. Annual values (average ± SD) were based on two calendar years (March 2011-February 2012 and March 2013-February 2014). Data for the third year (March 2012-February 2013) were excluded because flooding caused incomplete data sets for South and North Kinangop (see Methods). Likewise, monthly values (average ± SD, range) were based on 24 months (March 2011- February 2012 and March 2013-February 2014).

Location	Annual biomass ground invertebrates log(pitfall)	Monthly biomass ground invertebrates log(pitfall)		Annual biomass flying invertebrates log(sweep-net session)	Monthly biomass flying invertebrates log(sweep-net session)	
	Mean ± SD	Mean ± SD	Range	Mean ± SD	Mean ± SD	Range
S. Kinangop	34.6 ± 1.74	2.88 ± 0.27^a^	2.26–3.30	15.7 ± 3.92	1.31 ± 0.65^a^	0.00–2.45
N. Kinangop	33.2 ± 0.96	2.77 ± 0.38^a^	2.06–3.65	12.4 ± 2.12	1.04 ± 0.56^a^	0.00–1.80
Kedong	35.6 ± 1.88	2.97 ± 0.41^a^	2.10–3.83	21.5 ± 3.21	1.79 ± 0.55^b^	0.00–2.69

Superscripts indicate subsets of significant differences (P<0.05) among locations in post-hoc tests, after mixed-model analyses.

Despite differences in climate and invertebrate biomass, Red-capped Larks bred in all three locations (Table 5). In the period January 2011 –March 2014, we found 74 nests in South Kinangop, 63 nests in North Kinangop and 153 nests in Kedong (Table 5). Calculating nest index corrected for search effort, we found the highest numbers in Kedong, followed by North Kinangop (63% lower than Kedong) and South Kinangop (84% lower than in Kedong).

**Table 5:** Total number of nests found, percentage successful nests, and annual and monthly nest index (number of nests/10 h search effort) in South Kinangop, North Kinangop and Kedong. Total number of nests found is based on the period January 2011 –March 2014. Nests failed due to a variety of reasons, including predation, abandonment, flooding and human destruction. Average nest indices are based on two complete calendar years (March 2011—February 2012, and March 2013 –February 2014; 24 months) and excluded the year in which flooding occurred (see Methods). Superscripts indicate subsets of significant differences (P<0.05) among locations in post-hoc tests, after mixed model analyses.

Location	Number of nest found (% successful)	Annual nest index (number of nests/10h of search effort)	Monthly nest index (number of nests/10h of search effort)	
		Mean ± SD (n = 2 years)	Mean ± SD (n = 24 months)	Range
S. Kinangop N. Kinangop Kedong	74 (12%) 63 (26%) 153 (18%)	1.5 ± 0.16 3.7 ± 1.91 10.0 ± 6.89	0.13 ± 0.23^a^ 0.31 ± 0.59^ab^ 0.83 ± 1.30^b^	0.00–0.86 0.00–2.61 0.00–5.28

### Year-round variation in environmental conditions, invertebrates and breeding of Larks

In all three locations, rainfall occurred in all calendar months of the year, but the amount of rainfall in any given month was highly variable and unpredictable among years (Figs 1A, 2A and 3A). The only consistent annually recurring observation was that January and February were dry in all three locations in all four years (with the exception of Kedong in 2014). Outside of this annually recurring dry season, there was no month without rain in North Kinangop and only one month was without rain in South Kinangop (March 2014). However, Kedong received no rain at all during six months in 2013 (June-November), in contrast to 2011 and 2012 when this location received rain every month. Average monthly T_min_ and average monthly T_max_ varied unpredictably throughout the year in all locations and years, but generally, T_min_ were lowest and T_max_ were highest in January and February each year (Figs 1B, 2B and 3B). Average monthly T_max_ varied between 21.2°C and 30.0°C in South Kinangop, between 22.1°C and 30.5°C in North Kinangop, and between 25.3°C and 34.9°C in Kedong (Table 3). Likewise, average monthly T_min_ varied between 3.0°C and 8.2°C in South Kinangop, between 3.0°C and 13.7°C in North Kinangop, and between 6.2°C and 15.7°C in Kedong (Table 3).

Ground and flying invertebrates were present in all months in all locations, but biomasses varied among months and among years in an unpredictable manner (Figs 1C, 2C and 3C).

Overall, we observed Red-capped Larks breeding in all calendar months, but they did not breed in every month in every year in any of the three locations (Figs 1A, 2A and 3A). In South Kinangop, we found nests in the calendar months January-April and June-August, and in 10 out of 30 months total (33%). In North Kinangop, we found nests in all calendar months except June and July, and in 21 out of 39 months total (54%). Finally, in Kedong, we found nests in all calendar months and in 20 out of 39 months total (51%). In all locations, year-to-year variation in nest index was present, with highest nest indices in 2012 (Figs 1A, 2A and 3A).

### Associations between nest index, environmental factors and invertebrate biomass

The abiotic (rainfall, T_min_ and T_max_) and biotic (ground and flying invertebrate biomass) factors, in the month before or the month of breeding, explained little variation in occurrence or intensity of breeding of Larks, in any of the three locations (Table 6). For each location, and for both sets of analyses, no single best model emerged. For the analysis of occurrence of breeding there were subsets of six (South Kinangop), 13 (North Kinangop), and four (Kedong) models with ΔAICc < 2 (Table 6A). These models contained zero to two of the ten abiotic and biotic factors included in the analysis; each of the subsets contained four to seven of the ten factors (Table 6A). The explained variation (D^2^) of models was consistently low, and for South Kinangop and North Kinangop, the model sets included the intercept-only “null” model (i.e., no environmental factors; Table 6A).

**Table 6:** Model selection results of (A) occurrence of breeding of Red-capped Larks in South Kinangop, North Kinangop and Kedong, and (B) intensity of breeding in North Kinangop and Kedong as a function of biotic and abiotic factors in the month prior to the breeding observation and the month of the breeding observation (see Methods for details). Parameters, degrees of freedom, AICc, ΔAICc, weights and the explained variation (D^2^ in (A) and R^2^ in (B)) of the top-model and models with a ΔAICc <2.

A	Model ranking	Parameters	DF	AICc	ΔAICc	Weight	D^2^
South Kinangop (n = 22 months)	1 2 3 4 5 6	Intercept Intercept +T_min_[prior] Intercept +Flying invertebrates[prior] Intercept +T_max_[prior] Intercept +T_max_[current] Intercept + Ground invertebrates[current]	2 3 3 3 3 3	32.1 33.1 33.6 33.6 33.8 34.1	- 0.98 1.43 1.47 1.71 1.96	0.296 0.181 0.145 0.142 0.126 0.111	0.00 0.08 0.06 0.06 0.04 0.03
North Kinangop (n = 27 months	1 2 3 4 5	Intercept +T_max_[current] Intercept +T_max_[prior] Intercept +T_min_[prior] Intercept +T_max_[current]+Flying invertebrates[prior] Intercept	3 3 3 4 2	39.9 40.0 40.2 40.2 40.2	- 0.07 0.26 0.31 0.34	0.119 0.115 0.105 0.101 0.101	0.15 0.15 0.15 0.23 0.00
	6 7 8 9 10 11 12 13	Intercept +T_max_[prior]+Flying invertebrates[prior] Intercept +T_max_[current]+Flying invertebrates[current] Intercept +T_min_[prior]+ Ground invertebrates[current] Intercept +Ground invertebrates[prior] Intercept +T_max_[current]+T_min_[prior] Intercept + Flying invertebrates[prior] Intercept +Ground invertebrates[current] Intercept +T_max_[prior]+T_min_[prior]	4 4 4 3 4 3 3 4	40.3 41.4 41.4 41.4 41.5 41.8 41.9 41.9	0.42 1.45 1.45 1.5 1.58 1.91 1.98 1.98	0.096 0.058 0.058 0.057 0.053 0.046 0.045 0.045	0.23 0.20 0.19 0.11 0.19 0.09 0.09 0.18
Kedong (n = 31 months)		Intercept + Ground invertebrates[current] Intercept + T_max_[prior]+Ground invertebrates[current] Intercept + T_max_[current]+ Ground invertebrates[current] Intercept +T_min_[prior]+ Ground invertebrates[current]	3 4 4 4	43.6 44.1 45.2 45.3	- 0.47 1.55 1.68	0.372 0.295 0.172 0.161	0.17 0.23 0.20 0.20
B	Model ranking	Parameters	DF	AICc	ΔAICc	Weight	R^2^
North Kinangop (n = 14 months)	1 2 3 4 5 6	Intercept +T_max_[current]+Ground invertebrates[prior] Intercept +T_max_[current] Intercept Intercept +T_max_[prior] Intercept +T_min_[prior]+Ground invertebrates[prior] Intercept +Ground invertebrates[prior]	4 3 2 3 4 3	14.1 14.9 15.1 15.4 15.7 15.9	- 0.73 1.00 1.25 1.59 1.77	0.270 0.188 0.164 0.145 0.122 0.112	0.35 0.16 - 0.13 0.27 0.10
Kedong (n = 17 months)	1 2 3 4	Intercept Intercept +T_min_[current] Intercept + Rainfall[prior] Intercept +T_max_[prior]	2 3 3 3	66.7 66.9 67.6 68.4	- 0.16 0.83 1.71	0.332 0.307 0.220 0.141	- 0.10 0.06 0.01

**Fig 4:**
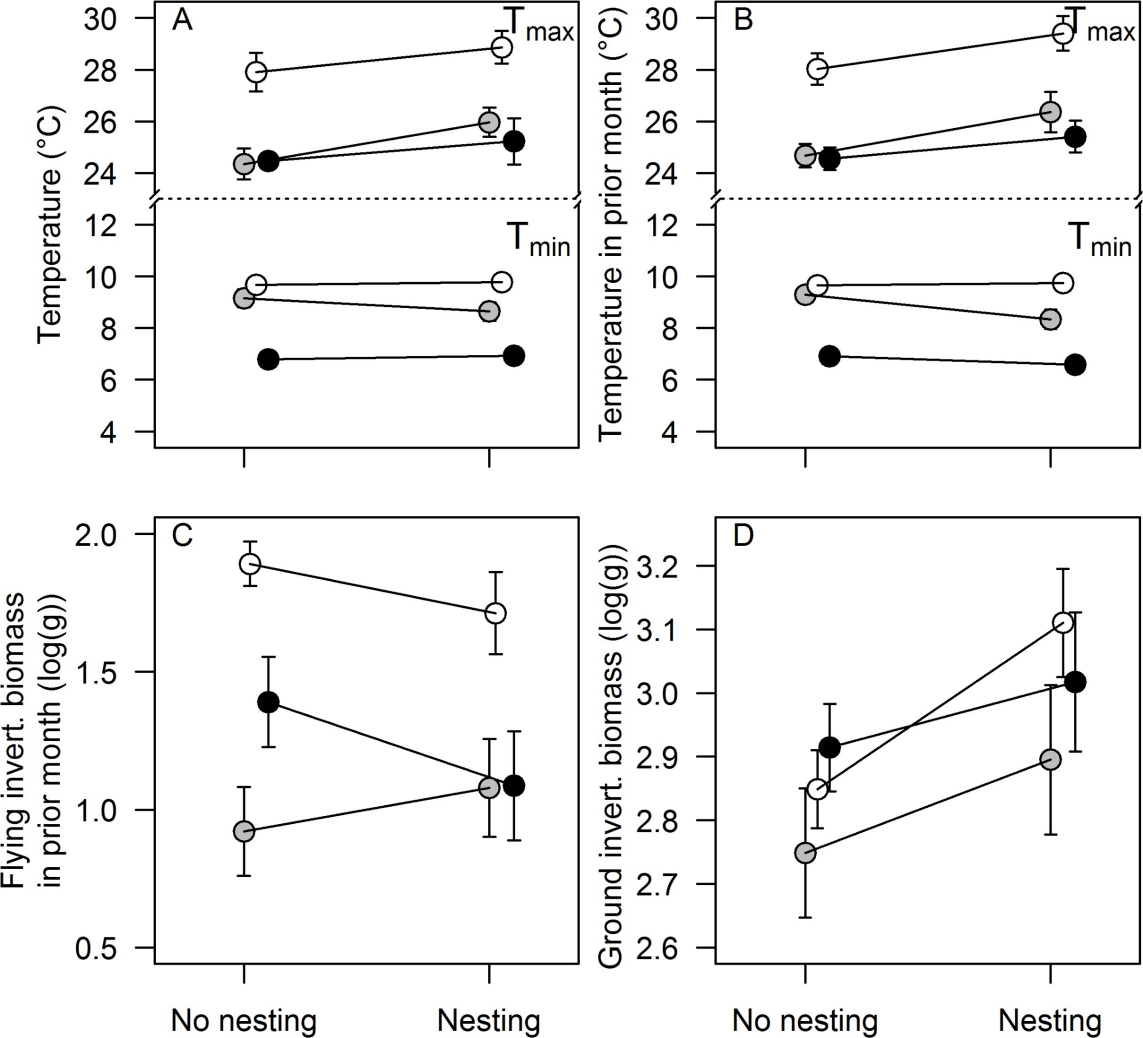
Average monthly (± SE) values during non-breeding and breeding months, for the biotic and abiotic factors that were selected in the model selection analysis (Table 6, Table 7) for South Kinangop (black symbols), North Kinangop (grey symbols), and Kedong (white symbols). A. Minimum (T_min_) and maximum (T_max_) temperature (°C) in current month, B. Minimum (T_min_) and maximum (T_max_) temperature (°C) in prior month, C. Flying invertebrate biomass (log(g dry weight)) in prior month. D. Ground invertebrates biomass (log(g dry weight)) in current month.

Using model averaging and standardization to explore the relative contribution of the environmental parameters to breeding, we could not identify one or more significant environmental factors that explained breeding in Larks (Table 7A; Fig 4A–4D).

**Table 7:** Model averaging and standardization results of occurrence of breeding of Red-capped Larks in South Kinangop, North Kinangop and Kedong, as a function of biotic and abiotic factors in the month prior to the breeding observation and the month of the breeding observation (see Methods for details). Standardized estimates (± SE, 95% confidence intervals) for biotic and abiotic factors of the average model per location are based on the model subsets in Table 6.

A	Parameter	Estimate	S.E.	95% CI
South Kinangop	Intercept	-0.81	0.50	-1.80–0.18
	T_max_[current]	0.12	0.12	-0.11–0.35
	T_max_[prior]	0.15	0.15	-0.14–0.44
	T_min_[prior]	-0.25	0.21	-0.67–0.16
	Ground invertebrates[current]	0.10	0.11	-0.13–0.32
	Flying invertebrates[prior]	-0.16	0.15	-0.45–0.13
North Kinangop	Intercept	-0.01	0.36	-0.73–0.70
	T_max_[current]	0.41	0.25	-0.09–0.90
	T_max_[prior]	0.33	0.23	-0.12–0.78
	T_min_[prior]	-0.26	0.19	-0.62–0.10
	Ground invertebrates[current]	0.07	0.07	-0.06–0.21
	Flying invertebrates[current]	0.04	0.04	-0.03–0.12
	Flying invertebrates[prior]	0.28	0.24	-0.19–0.76
Kedong	Intercept	0.33	0.43	-0.51–1.18
	T_max_[current]	0.02	0.02	-0.02–0.06
	T_max_[prior]	0.08	0.05	-0.03–0.18
	T_min_[prior]	-0.14	0.15	-0.44–0.15
	Ground invertebrates[current]	2.37	1.19	0.04–4.71
B				
North Kinangop	Intercept	0.553	0.09	0.38–0.72
	T_max_[current]	0.168	0.08	0.02–0.32
	T_max_[prior]	0.046	0.03	-0.01–0.10
	T_min_[prior]	-0.042	0.02	0.08–0.00
	Ground invertebrates[prior]	-0.168	0.09	-0.34–0.00
Kedong	Intercept	1.656	0.36	0.94–2.37
	Rainfall[prior]	0.234	0.16	-0.09–0.56
	T_max_[prior]	-0.117	0.11	-0.33–0.09
	T_min_[current]	-0.37	0.22	-0.81–0.07

For the analysis of intensity of breeding there were subsets of six (North Kinangop) and three models (Kedong) with AIC_c_ < 2 (Table 6B). These models contained zero to two of the ten abiotic and biotic factors included in the analysis; each of the subsets contained two to seven of the ten factors (Table 6B). The explained variation (R^2^) of models was consistently low, and for North Kinangop, the model sets included the intercept-only “null” model (i.e., no environmental factors; Table 6B). Using model averaging and standardization to explore the relative contribution of the environmental parameters to breeding, we could only identify one significant environmental factor that explained breeding in Larks (i.e. T_max_ in North Kinangop; Table 7B).

## Discussion

This study showed that year-round breeding activities of Red-capped Larks in three climatically-distinct equatorial populations were not associated with rainfall, temperature and invertebrate biomass, across and within locations. Across locations that represent a gradient of rainfall and temperature, we found no support for the prediction that drier and warmer locations had lower invertebrate biomass and less breeding activity of Larks. In line with these results, within each location we also found no evidence that breeding was timed to co-occur with rainfall, higher or lower temperatures or invertebrate biomass. Instead, we observed highly unpredictable and irregular variation in environmental variables, invertebrate biomass and breeding of Larks, among months and among years. Red-capped Larks bred in all calendar months overall, but they did not breed in every month in every year in every location. Our findings raise the question of which factors do trigger equatorial Red-capped Larks to initiate breeding. They suggest that environmental conditions vary at a small spatio-temporal scale and often provide minimum requirements for breeding, at least for some individuals in each population.

Despite the small geographical distances (19–34 km) separating the three locations, our weather data allowed us to quantitatively confirm the distinct spatial patterns in rainfall and temperatures that we expected among locations due to orography (Table 1). However, against our predictions, the warmest and driest location, Kedong, had the highest flying invertebrate biomasses and the highest Lark nest index. In contrast, the cool and wet climatic extreme, South Kinangop, had intermediate ground and flying invertebrate biomasses and the lowest Lark nest index. Overall, the general global trend of lower primary productivity in more arid environments [38, 30, 27] is not reflected in our invertebrate and Lark breeding data.

Other factors that, in combination with rainfall and temperature, may affect Lark ecology and that differ among locations include soil type, land use, and the occurrence of climatic excesses. South Kinangop’s climate and fertile soil allow for intensive crop cultivation, but the combination of heavy rains and poor soil drainage also make the area prone to flooding. Seminis, a study site in South Kinangop with communally grazed land, experiences multiple months of standing water in some years, which might negatively affect productivity and diversity of plants and invertebrates [39]. In contrast, Kedong’s grasslands are extensively grazed by diverse wild and domestic herbivores, a process that might increase primary productivity and favor invertebrate and Lark reproduction [40, 41, 27, 42].

All three locations showed a lack of predictable intra-annual patterns in rainfall, temperature and invertebrate biomasses. Lark breeding was most unpredictable in Kedong where we observed breeding in 51% of the months but in all 12 calendar months, and most predictable in South Kinangop, where breeding occurred in 33% of the months restricted to seven calendar months. In addition, variation among years contributed to the unpredictability in all locations. Although we did not identify relationships among rainfall and breeding during the 39 months of our study, we did observe that breeding activities were affected by multi-month rainfall excesses: during a six-month drought in Kedong breeding was almost (but not fully) absent, while extended flooding prevented birds from breeding in South Kinangop and destroyed nests in both South and North Kinangop. These observations are in line with other studies in highly variable and unpredictable environments that also show that flooding from heavy rainfall disrupts breeding by destroying nests, by directly killing eggs and young [28, 43], and by reducing food availability or foraging efficiency [28, 29, 10, 39]. Moreover, drought can result in food and foraging limitations, and birds may avoid breeding during dry seasons [12, 20, 44, 10, 45, 46, 23].

The results of our model selection analysis, which did not strongly identify any of the environmental factors as relevant for Lark breeding, have two potential explanations. Either the spatial scale at which we sampled rainfall, temperature, and invertebrates was not the scale that Red-capped Larks used to time breeding, or Red-capped Larks used other factors to initiate breeding. We sampled rainfall and temperature with one weather station per location, placed centrally among the multiple plots within a location but as a result also at varying distance to each plot (Table 1). Likewise, we sampled invertebrates at only one transect in each plot. One might wonder if the small-scale spatio-temporal variation in environmental and ecological factors that we discovered calls for measuring rainfall, temperature and invertebrates at the level of Lark territories. While the addition of territory-level data might be interesting, we do not believe that sampling scale underlies our population-level findings about Lark breeding. Moving around in small flocks when not breeding, Red-capped Larks do not appear to stay within their breeding territory year-round (pers. obs. based on color-ringed individuals), a behavior that contrasts with some other Lark species (e.g., Hoopoe Larks, *Alaemon alaudipes*, in the Arabian Desert [45].

We therefore propose that Red-capped Larks use other factors to time reproduction, with prime candidates being nest predation [47, 48, 49, 50, 51], female protein reserves [52, 53, 54, 55], or social factors. Although a detailed study remains to be done, nest predation in our study sites is generally high, with only 53 nests that fledged from the total of 290 nests found in different stages of the nesting cycle (pers. obs.). In the face of such intense nest predation Red-capped Larks may breed opportunistically [11, 56, 57, 45, 42]. If environmental conditions are always permissive, birds may breed whenever they have resupplied their reserves after a failed nest attempt [58]. This would also be in line with earlier studies on equatorial passerines with opportunistic breeding schedules, in which the authors suggested that protein reserves of individual females may determine whether and when they breed, leading to asynchronous year-round breeding activities at the population level [52, 53, 54, 55, 56]. Social factors can affect breeding decisions in multiple ways. For example, some individuals may time their breeding to benefit from peak food availability [19, 25, 2, 14], while others may avoid competition for food or nesting space by conspecifics or other species by choosing “unpopular” times between food peaks [59]. In addition, timing of breeding may be influenced by predation-avoidance strategies (i.e., a pair avoids being the only one breeding at a particular time due to the high predation risk associated with that position [60, 61] and prolonged nestling dependence on parents [62]. A better understanding of these possible mechanisms will come from studies at the individual level that complement our population level findings.

## Supporting information

S1 Appendix Number of samples (n) and range of length (minimum L in mm, maximum L in mm) and width (minimum W in mm, maximum W in mm) of invertebrates used in generating calibration curves, and statistics per curve (coefficients a, b, c; adjusted r^2^, degrees of freedom, F and P-values), per invertebrate category we used to predict body mass from length and width. Calibration curves were fit with the formula log(biomass in mg dry weight) = log(*a*) + *b* log(length in mm) + *c* log (width in mm), where *a*, *b* and *c* are coefficients of the model (see Methods).
(DOCX)

S1 File Outcome of the investigation of the effect of maximum temperature, minimum temperature, rainfall, ground and flying invertebrates on nest index at time windows and time lags up until 6 month prior to the current month in Red-capped Larks living in three climatically-distinct Kenya locations, South Kinangop, North Kinangop and Kedong during March 2011 –February 2014. Please note the inclusion of Table A and Figure A in S2 File.
(DOCX)

S1 Table Correlation coefficients (below the diagonal) and P-values (above the diagonal) of pairwise correlations among the 3 weather variables and 2 invertebrate variables in three Kenya locations, South Kinangop, North Kinangop and Kedong during March 2011 –February 2014. (DOCX)

S2 Table Coefficients of variation of monthly (n = 36 months) rainfall, minimum temperature and maximum temperatures as measured by our weather stations in South Kinangop, North Kinangop and Kedong, during March 2011 –February 2014. F-tests of equal variances indicated that there were no significant differences among locations (P<0.05; indicated by superscripts).
(DOCX)
