# Supplementary material for: Year-round breeding equatorial Larks from three climatically-distinct populations do not use rainfall, temperature or invertebrate biomass to time reproduction
Source: PLoS One. 2017 Apr 18;12(4):e0175275. doi: 10.1371/journal.pone.0175275 (PMC5395156; doi:10.1371/journal.pone.0175275)
Supplement: S1 Appendix — Calibration curves were fit with the formula log(biomass in mg dry weight) = log(a) + b log(length in mm) + c log (width in mm), where a, b and c are coefficients of the model (see Methods). (DOCX) [file pone.0175275.s001.docx]

| Insect group | n | Min L (mm) | Max L (mm) | Min W (mm) | Max W (mm) | a ± SE | b ± SE | c ± SE | Adj. r^2^ | d.f. | F | P-value |
| --- | --- | --- | --- | --- | --- | --- | --- | --- | --- | --- | --- | --- |
| Ants | 210 | 1.9 | 8.4 | 0.1 | 3.0 | 0.002±1.2785 | 3.70±1.486 | 0.12±1.425 | 0.84 | 2, 207 | 551.9 | <0.001 |
| Bees and wasps | 157 | 3.0 | 17.0 | 1.0 | 6.3 | 0.088+1.2612 | 1.84±1.451 | 0.8±1.383 | 0.82 | 2, 154 | 351 | <0.001 |
| Beetles and bugs | 224 | 3.8 | 31.5 | 1.6 | 12.9 | 0.094±1.178 | 1.36±1.413 | 1.56±1.369 | 0.95 | 2, 221 | 2031 | <0.001 |
| Butterflies and moths | 121 | 4.3 | 28.4 | 0.9 | 6.0 | 0.006±1.3446 | 2.92±1.383 | 0.32±1.399 | 0.86 | 2, 118 | 377.3 | <0.001 |
| Caterpillars, caddisflies, stoneflies | 38 | 5.8 | 43.4 | 0.9 | 7.0 | 0.031±2.7625 | 2.1±3.009 | 0.31±2.834 | 0.69 | 2, 35 | 38.11 | <0.001 |
| Diplura, millipede, centipede and earthworms | 129 | 5.1 | 105.0 | 0.3 | 4.0 | 0.074±1.3747 | 1.63±1.273 | 0.73±1.428 | 0.83 | 2, 125 | 315.8 | <0.001 |
| Flies | 381 | 2.0 | 22.0 | 0.2 | 5.5 | 0.101±1.1592 | 1.56±1.272 | 0.68±1.213 | 0.79 | 2, 378 | 705.9 | <0.001 |
| Grasshoppers, crickets and mantises | 219 | 4.7 | 54.6 | 0.8 | 44.9 | 0.144±1.2515 | 1.62±1.373 | 0.88±1.386 | 0.84 | 2, 216 | 556.9 | <0.001 |
| Spiders, ticks and mites | 228 | 2.3 | 12.9 | 1.3 | 7.4 | 0.087±1.225 | 1.98±1.424 | 0.78±1.378 | 0.72 | 2, 225 | 296.5 | <0.001 |
| Rest category (woodlice, cicadas, cockroaches and earwigs) | 114 | 2.3 | 19.9 | 0.5 | 5.5 | 0.126±1.211 | 1.40±1.308 | 1.15±1.282 | 0.92 | 2, 111 | 626.5 | <0.001 |
