# Supplementary material for: Year-round breeding equatorial Larks from three climatically-distinct populations do not use rainfall, temperature or invertebrate biomass to time reproduction
Source: PLoS One. 2017 Apr 18;12(4):e0175275. doi: 10.1371/journal.pone.0175275 (PMC5395156; doi:10.1371/journal.pone.0175275)

Introduction

Our multivariate approach in the main text did not allow investigation of more than two time periods (current and prior months). We therefore analyzed the data again, using the R-package *climwin* (version 1.0.0; Bailey & van de Pol 2016; see van de Pol *et al.* 2016). We investigated whether single variables (maximum temperature, minimum temperature, rainfall, flying and ground invertebrates) at time windows and time lags up until 6 month prior to the current month had an effect on nest index of Red-capped Larks in Kenya. Because this R-package does not allow investigation of multiple environmental variables at the same time, we preferred to use the approach of only looking at 2 time lags (current and prior) in the main text.

Methods

The function *slidingwin* in the R-package *climwin* allowed identification of the best time window (considering a single month, or the mean of multiple months) and time lag (current month or a given number of months prior) in which environmental factors best predicted nest index, going back multiple months. Nest index was calculated as number of nests found in 10 search hours (see main text). In at least 46% of the studied months nest index was zero (no breeding; South Kinangop 20 of 30 months, North Kinangop 18 of 39 months, Kedong 19 of 39 months). Therefore we analyzed the data in two steps: first we transformed the data into a binomial parameter (0 = no breeding, 1 = breeding) to investigate the influence of environmental factors on occurrence of breeding. We used generalized linear models with a binomial distribution for these analyses. Subsequently we analyzed a subset of the data where nest index was larger than 0. We used these data to investigate the influence of environmental factors on intensity of breeding, given that birds were breeding. We used linear models with a Gaussian distribution for these analyses.

For the weather variables we used a maximum time lag of 6 months prior to the current month, and for the invertebrate variables a maximum time lag of 4 months prior to the current month (because of more missing values). For both the weather and the invertebrate variables we used a minimum time lag of 0 (current month). Using the function *slidingwin*, we ran all possible models and calculated the difference in AICc with a null-model which contained no environmental factors (ΔAICc). A positive ΔAICc indicated that the considered model is worse than the null-model. The “best model” was the model with the lowest ΔAICc. Because we ran multiple models (28 for the weather variables and 15 for the invertebrate variables), we did a randomization (1000 times) for each variable, to calculate how high the chance was of finding a ΔAICc value of the best model, thus calculating the significance of a model. We also determined how many models had to be included to cover 95% of the model weights (i.e. the 95% CI of models) and whether this included a model with current or prior months. This indicated how many models were alternatively good models. It also showed whether 95% CI included the models that tested the variables in the prior and current month (i.e. months that were tested in the multivariate analysis in the main text). This way we could determine what the best time window was for each variable, and whether environmental factors were likely to be important for determining breeding of Red-capped Larks. We did these analyses for the three locations separately. Because month was not important as random effect in the original analysis (AICc values were always higher for models without random effect), and we aimed to maximize statistical power, we decided not to take into account month as random effect.

Results

Determinants of occurrence of breeding in Red-capped Larks

In each location, only one environmental variable- a different one in each location- significantly affected whether Red-capped Larks bred or not (Table S1A): in North Kinangop minimum temperature averaged over 3 months prior to current month; in South Kinangop ground invertebrates averaged over 3 to 2 months prior to current month, and in Kedong rainfall averaged over 5 months prior to current month. In all three cases 95 % CI of the model weight comprised at least 33 % of the total models (North Kinangop: 13 out of 28 models, South Kinangop: 5 of 15 models, Kedong: 10 of 28 models). The environmental factors that were tested in the main text (current and prior month) were almost always included in the 95% CI of the model weights. We found two exceptions: 1. The 95% CI of models of rainfall in Kedong did not include the models with only prior or current month. 2. The 95% CI of models of ground invertebrates in South Kinangop also did not include the models with only prior or current month. The sample size for months in which we obtained ground invertebrate data, and birds were breeding (i.e. occurrence of breeding = 1) was low in South Kinangop (n=5), and this caused the reliability of the latter results to be questionable.

Determinants of intensity of breeding in Red-capped Larks

Only in South Kinangop did one environmental factor significantly affect intensity of breeding in Red-capped Larks (Table S1B): maximum temperature in 2 months prior to the current month. For this parameter 95 % CI of the model weight comprised only 14% of the total models (4 out of 28 models). The environmental factors that we tested in the main text (current and prior month) were almost always included in the 95% CI of the model weights. We found two exceptions: The 95 % CI of models of maximum temperature and ground invertebrates in South Kinangop. The latter was not significant.

Conclusion

Considering weather and invertebrate factors that preceded breeding by up to six months , we found no clear evidence that a certain time window or time lag was important for occurrence of breeding in any of the three study locations. In 13 out of the 15 sets of analyses of breeding occurrence (Table S1A), the models that included current or prior month were covered within the 95% CI set of models weights. This is relevant because current and prior month were included in the multivariate analyses in the main text. For the intensity of breeding, only one environmental factor (maximum temperature 2 months prior in South Kinangop) appeared to be important. Again in 13 out of the 15 sets of analyses of breeding intensity (Table S1B), the models that included current and prior month were covered within the 95% CI set of model weights. Of these, in total, four cases in which the models with current and/or prior month fell outside the 95% CI of model weights, only one case had a relatively narrow CI: the 95% CI for the model with maximum temperature 2 months prior in South Kinangop explaining the intensity of breeding comprised only 4 out of 28 model weights. However, when inspecting the relationship between maximum temperature 2 months prior and breeding intensity in South Kinangop we found that it was highly dependent on only two or three data points (figure 1S). Therefore, our general conclusion is that there is no clear evidence for any of the environmental factors to be important predictors for occurrence or intensity of breeding of Red-capped Larks when evaluating periods of up to six months before a breeding event. In combination with the results from the multivariate analyses presented in the main text, we conclude that no matter how we analyze our data sets, Red-capped Larks time their breeding independent of weather and invertebrate availability.

Bailey, L.D. & Van de Pol, M. (2016) Climwin: c*limate Window Analysis*. URL http://cran.r-project.org/web/packages/climwin/index.html.

Van de Pol, M., Bailey, L.D., McLean, N., Rijsdijk, L., Lawson, C.R. & Brouwer, L. (2016) Identifying the best climatic predictors in ecology and evolution. *Methods in Ecology and Evolution*, **7**, 1246–1257.

Table A in S2 File: Results of the analyses on environmental factors on (A) occurrence of breeding and (B) and intensity of breeding in Red-capped Larks in North Kinangop, South Kinangop and Kedong. The analyses considered a window of 6 to 0 (abiotic variables), or 4 to 0 (biotic variables) months prior to the month of breeding. Presented are: the best climatic window, ΔAICc in comparison to a model without the parameter, the P-value of the best model, the number of models that comprise 95% of the model weights, total number of models, whether 95% model weights included the current or prior models (that we used in the main text), sample size (in (A) given separately for nest index=0 and nest index =1) and the coefficient ± standard error of the best model.

| A |  |  |  |  |  |  |  |  |  |
| --- | --- | --- | --- | --- | --- | --- | --- | --- | --- |
| Area | Parameter | time window | ΔAICc | P_AICc_ | No. of models in 95% weight | Total No. of models | Prior or current in 95% weights | Sample size (0/1) | Beta (S.E.) |
| North Kinangop | Rainfall | 3 - 1 | -4.36 | 0.15 | 19 | 28 | yes | 16/17 | -0.05 (0.04) |
|  | Maximum temperature | 1 - 0 | -3.44 | 0.19 | 22 | 28 | yes | 16/17 | 0.44 (0.21) |
|  | Minimum temperature | 3 - 0 | -8.45 | 0.03 | 13 | 28 | yes | 16/17 | -1.10 (0.42) |
|  | Flying invertebrates | 2 - 1 | 0.27 | 0.64 | 13 | 15 | yes | 13/7 | -0.65 (0.48) |
|  | Ground invertebrates | 0 | -0.03 | 0.57 | 13 | 15 | yes | 11/6 | 1.00 (0.68) |
| South Kinangop | Rainfall | 4 | -6.04 | 0.10 | 17 | 28 | yes | 16/8 | 0.03 (0.02) |
|  | Maximum temperature | 3 - 0 | -1.23 | 0.42 | 24 | 28 | yes | 16/8 | 0.68 (0.38) |
|  | Minimum temperature | 5 - 3 | -2.92 | 0.28 | 22 | 28 | yes | 16/8 | 1.65 (0.81) |
|  | Flying invertebrates | 2 - 1 | -1.12 | 0.44 | 13 | 15 | yes | 16/7 | -0.80 (0.48) |
|  | Ground invertebrates | 3 - 2 | -10.68 | 0.02 | 5 | 15 | no | 11/5 | 19.00 (12.01) |
| Kedong | Rainfall | 5 - 0 | -13.17 | 0.002 | 10 | 28 | no | 17/16 | 0.11 (0.04) |
|  | Maximum temperature | 6 - 0 | -3.43 | 0.15 | 24 | 28 | yes | 17/16 | 0.71 (0.32) |
|  | Minimum temperature | 2 - 1 | 0.73 | 0.90 | 26 | 28 | yes | 17/16 | -0.36 (0.30) |
|  | Flying invertebrates | 4 | -4.32 | 0.13 | 12 | 15 | yes | 8/11 | -2.09 (1.14) |
|  | Ground invertebrates | 1 | 1.00 | 0.78 | 14 | 15 | yes | 8/16 | -0.65 (0.57) |
| B |  |  |  |  |  |  |  |  |  |
| Area | Parameter | time window | ΔAICc | P_AICc_ | Nr. of models in 95% weight | Total nr. of models | Prior or current in 95% weights | Sample size | Beta (S.E.) |
| North Kinangop | Rainfall | 5 | 0.02 | 0.66 | 25 | 28 | yes | 17 | 0.01 (0.01) |
|  | Maximum temperature | 3 - 0 | -3.77 | 0.10 | 19 | 28 | yes | 17 | 0.21 (0.08) |
|  | Minimum temperature | 3 - 0 | -5.11 | 0.10 | 20 | 28 | yes | 17 | 0.31 (0.10) |
|  | Flying invertebrates | 1 | 2.76 | 0.40 | 13 | 15 | yes | 7 | -0.06 (0.03) |
|  | Ground invertebrates | 4 - 2 | 5.72 | 1.00 | 12 | 15 | yes | 6 | 0.32 (0.15) |
| South Kinangop | Rainfall | 6 - 4 | 0.51 | 0.62 | 23 | 28 | yes | 8 | 0.06 (0.02) |
|  | Maximum temperature | 2 | -10.80 | 0.003 | 4 | 28 | no | 8 | 1.45 (0.23) |
|  | Minimum temperature | 4 - 0 | -6.21 | 0.08 | 11 | 28 | yes | 8 | -6.39 (1.42) |
|  | Flying invertebrates | 3 - 0 | 5.08 | 1.00 | 5 | 15 | yes | 7 | -1.14 (0.91) |
|  | Ground invertebrates | 2 | 1.88 | 0.86 | 1 | 15 | no | 5 | 32.58 (3.12) |
| Kedong | Rainfall | 1 | 1.26 | 0.88 | 26 | 28 | yes | 16 | 0.01 (0.01) |
|  | Maximum temperature | 1 | 1.71 | 0.84 | 26 | 28 | yes | 16 | -0.15 (0.14) |
|  | Minimum temperature | 0 | 1.65 | 0.96 | 26 | 28 | yes | 16 | -0.24 (0.21) |
|  | Flying invertebrates | 4 | 2.44 | 0.99 | 14 | 15 | yes | 11 | -0.48 (0.46) |
|  | Ground invertebrates | 3 | 1.10 | 0.70 | 14 | 15 | yes | 16 | -0.52 (0.32) |

Fig A in S2 File: the relationship between maximum temperature of 2 months prior to the current month and intensity of breeding (nest index) in South Kinangop.


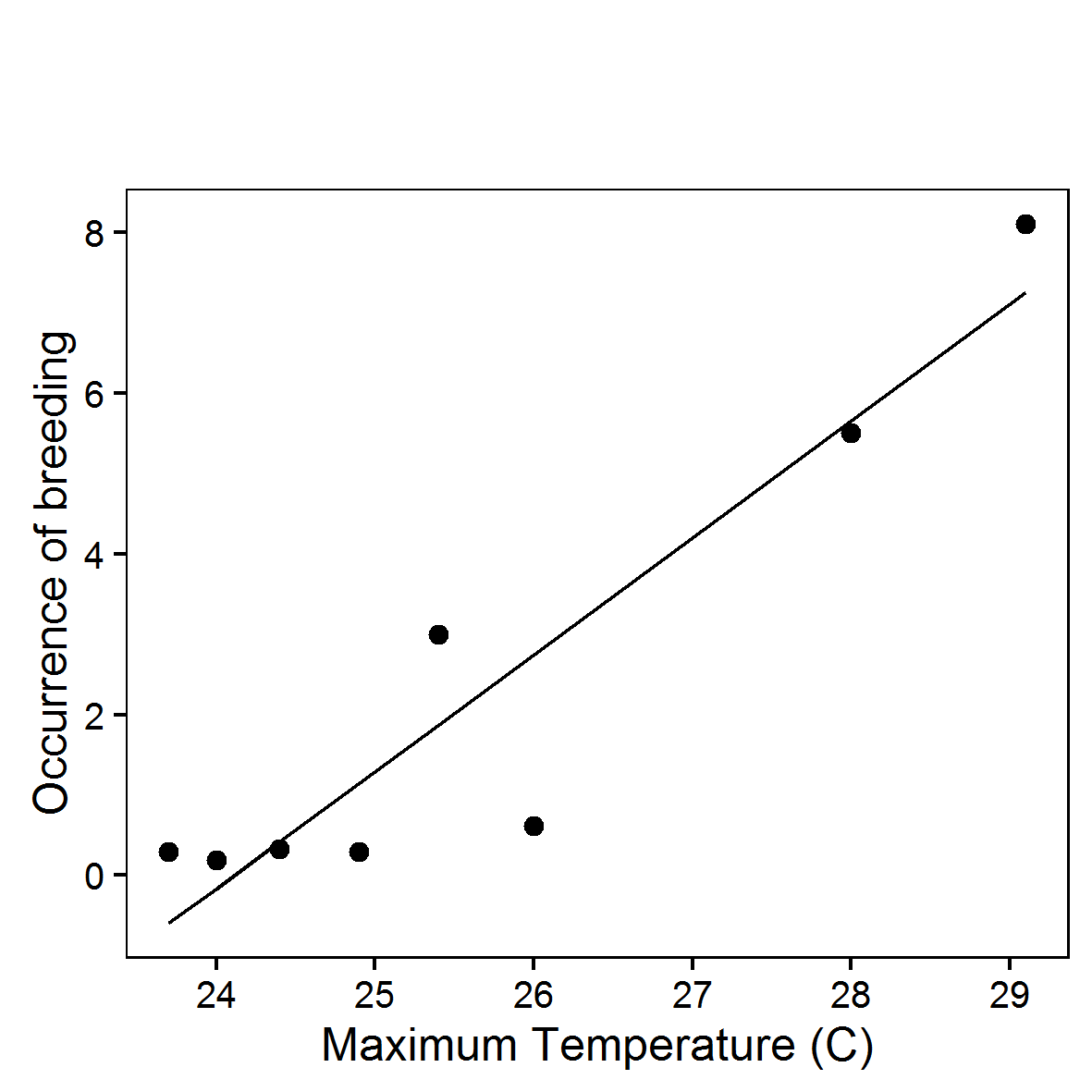

Supplement: S1 File — Please note the inclusion of Table A and Figure A in S2 File. (DOCX) [file pone.0175275.s002.docx]
