# Supplementary material for: Year-round breeding equatorial Larks from three climatically-distinct populations do not use rainfall, temperature or invertebrate biomass to time reproduction
Source: PLoS One. 2017 Apr 18;12(4):e0175275. doi: 10.1371/journal.pone.0175275 (PMC5395156; doi:10.1371/journal.pone.0175275)
Supplement: S1 Table — (DOCX) [file pone.0175275.s003.docx]

|  | Rainfall | Tmax | Tmin | Ground invertebrates | Flying invertebrates |
| --- | --- | --- | --- | --- | --- |
| South Kinangop (n=22 months) | |  |  |  |  |
| Rainfall |  | 0.20 | <0.001 | 0.73 | 0.79 |
| Tmax | -0.28 |  | 0.12 | 0.16 | 0.03 |
| Tmin | 0.71 | -0.34 |  | 0.87 | 0.27 |
| Ground invertebrates | 0.08 | 0.31 | -0.04 |  | 0.96 |
| Flying invertebrates | -0.06 | -0.45 | 0.25 | -0.01 |  |
| North Kinangop (n=27 months) | |  |  |  |  |
| Rainfall |  | 0.07 | 0.01 | 0.54 | 0.06 |
| Tmax | -0.36 |  | 0.01 | 0.58 | 0.21 |
| Tmin | 0.49 | -0.52 |  | 0.63 | 0.94 |
| Ground invertebrates | 0.12 | 0.11 | 0.10 |  | 0.49 |
| Flying invertebrates | 0.36 | -0.25 | -0.02 | -0.14 |  |
| Kedong (n=31 months) | |  |  |  |  |
| Rainfall |  | 0.07 | 0.004 | 0.93 | 0.45 |
| Tmax | -0.33 |  | 0.01 | 0.95 | 0.39 |
| Tmin | 0.50 | -0.48 |  | 0.27 | 0.71 |
| Ground invertebrates | -0.02 | -0.01 | 0.21 |  | 0.004 |
| Flying invertebrates | -0.14 | 0.16 | 0.07 | -0.50 |  |
