# Supplementary material for: Year-round breeding equatorial Larks from three climatically-distinct populations do not use rainfall, temperature or invertebrate biomass to time reproduction
Source: PLoS One. 2017 Apr 18;12(4):e0175275. doi: 10.1371/journal.pone.0175275 (PMC5395156; doi:10.1371/journal.pone.0175275)
Supplement: S2 Table — F-tests of equal variances indicated that there were no significant differences among locations (P<0.05; indicated by superscripts). (DOCX) [file pone.0175275.s004.docx]

|  | Rainfall | Tmin | Tmax |
| --- | --- | --- | --- |
| South Kinangop | 89.36^a^ | 19.27^a^ | 8.46^a^ |
| North Kinangop | 72.04^a^ | 26.59^a^ | 8.94^a^ |
| Kedong | 112.00^a^ | 18.29^a^ | 8.53^a^ |
